# Effect of chaetocin on renal cell carcinoma cells and cytokine-induced killer cells

**DOI:** 10.3205/000231

**Published:** 2016-04-18

**Authors:** Roman Rombo, Hans Weiher, Ingo G.H. Schmidt-Wolf

**Affiliations:** 1 Center for Integrated Oncology (CIO), University of Bonn, Bonn, Germany; 2 Hochschule Bonn-Rhein-Sieg, Rheinbach, Germany

**Keywords:** clear cell renal cell carcinoma, chaetocin, cytokine-induced killer cells, CIK cells

## Abstract

We examined the cytotoxic effects of chaetocin on clear cell renal cell carcinoma (ccRCC) cells and the possibility to combine the effects of chaetocin with the effects of cytokine-induced killer cells (CIK) assayed by MTT assay and FACS analysis. Chaetocin is a thiodioxopiperazine produced by fungi belonging to the chaetomiaceae family. In 2007, it was first reported that chaetocin shows potent and selective* ex vivo *anti-cancer activity by inducing reactive oxygen species. CIK cells are generated from CD3+/CD56- T lymphocytes with double negative CD4-/CD8- phenotype that are isolated from human blood. The addition of distinct interleukins and antibodies results in the generation of CIK cells that are able to specifically target and destroy renal carcinoma cells. The results of this research state that the anti-ccRCC activity of chaetocin is weak and does not show a high grade of selectivity on clear cell renal cell carcinoma cells. Although the CIK cells show a high grade of selective anti-ccRCC activity, this effect could not be improved by the addition of chaetocin. So chaetocin seems to be no suitable agent for specific targeting ccRCC cells or for the combination therapy with CIK cells in renal cancer.

## Zusammenfassung

Wir untersuchten die zytotoxischen Effekte von Chaetocin auf Zellen von klarzelligem Nierenzellkarzinom (ccRCC) und die Möglichkeit, die Effekte von Chaetocin mit den Effekten von Zytokin-induzierten Killerzellen (CIK) zu kombinieren mit Hilfe von MTT Assay und FACS-Analyse. Chaetocin ist ein Thiodioxopiperazin hergestellt von Pilzen aus der Familie der Chaetomiaceae. In 2007 wurde erstmals berichtet, dass Chaetocin potente und selektive *ex vivo* Anti-Krebs-Aktivität durch Induzierung von reaktivem Sauerstoff besitzt. CIK-Zellen wurden aus CD3+/CD56- T-Lymphozyten aus menschlichem Blut hergestellt mit negativem CD4-/CD8- Phänotyp. Die Addition von bestimmten Interleukinen und Antikörpern führte zu der Herstellung von CIK-Zellen mit spezifischer Ausrichtung auf Nierenzellkarzinomzellen. Die Ergebnisse besagen, dass die anti-ccRCC Aktivität von Chaetocin schwach ist und keinen hohen Grad an Selektivität bzgl. klarzelligen Nierenzellkarzinomzellen besitzen. Obwohl CIK-Zellen einen hohen Grad an selektiver anti-ccRCC Aktivität besitzen, konnte dieser Effekt nicht durch die Zugabe von Chaetocin gesteigert werden. Daher erscheint Chaetocin keine geeignete Substanz für ein spezifisches Targeting von ccRCC-Zellen oder für die Kombinationstherapie mit CIK-Zellen beim Nierenzellkarzinom zu sein.

## Introduction

There are three major types of renal cell carcinoma (RCC). The most common types are clear cell renal cell carcinoma (ccRCC) (80–90%), papillary renal cell carcinoma (10–15%) and chromophobe renal cell carcinoma (3–5%) [1], [2].

Mutations of the epithelial cells lining the proximal tubule of the nephrons of the kidney are thought to give rise to RCC [3].

A suitable medical agent should be able to not only decrease the RCC growth but also kill specific cancerous cells without causing high casualties among non-cancerous cells. A promising new agent for targeting tumor cells is chaetocin.

Chaetocin was found to have a potent and selective *ex vivo* cytotoxic activity against different cancerous cell lines, however, the exact mechanism of its cytotoxic effects are still not fully understood. 

Most likely chaetocin is taken up by glutathione receptors followed by accumulation inside the cells. Hereby the drug accumulates in non-cancerous as well as in cancerous cells while the cytotoxic effects were mainly observed in cancerous cells. After the accumulation inside the cells chaetocin is thought to induce reactive oxygen species (ROS) that then kill the cancerous cells via oxidative stress but mostly spare the non-cancerous cells because they counter the oxidative stress with different enzymatic systems [4], [5]. The effects of chaetocin on cytokine-induced killer (CIK) cells is examined as well in order to determine if the CIK cells can be used along with chaetocin for ccRCC treatment.

CIK cells are cytotoxic lymphocytes resulting from treatment of peripheral blood mononuclear cells (PBMCs) that are isolated from whole blood and incubated with anti-CD3-antibody (α-CD3), interferon human interleukin 2 (IL-2) and human interleukin 1β (IL-1β) [6]. The treatment results in a new cell type with a unique receptor repertoire. CIK cells express the T-cell receptor CD3 as well as the natural killer cell receptor NKG2D that is thought to be responsible for the specific targeting of tumor cells [7], [8]. This recognition system is unlike the T cell receptor complex MHC unrestricted and specific for tumor cells. The mechanism behind the destruction of tumor cells is still unknown besides the fact that CIK cells produce a variety of cytokines that activate and influences other immune effector cells nearby. 

The aim of this thesis is to research the effects of chaetocin on ccRCC in comparison to non-cancerous cells and CIK cells in order to determine whether the medical agent can be used solely or in combination with CIK cells to increase the specific destruction of ccRCC cells while sparing non-cancerous cells.

## Material and methods

### Cell lines and culture conditions

For the cultivation of A-498, CAKI-2 and CCD-18Co (DMSZ, Braunschweig, Germany), 10 ml RPMI 1640 medium with L-glutamine and 2.0 g l^–1^ sodium bicarbonate (NaHCO_3_) (PAN BIOTECH, Aidenbach, Germany) containing 10% fetal bovine serum (FBS) (Gibco Life Technologies, Darmstadt Germany) and 100 U ml^–1^ penicillin and streptomycin (P/S) (Life Technologies, Darmstadt Germany) in a 75 cm^2^ culture flask was used. The cell lines were passaged every third or fourth day and stored in an incubator at 37°C in an atmosphere containing 95% air and 5% CO_2_.

Passaging was done by using 2 to 3 ml trypsin-EDTA (1x) (Gibco by Life Technologies, Darmstadt, Germany) after washing the cells with PBS (1x) (phosphate-buffered saline). Splitting in a ratio of 1/5 to 1/10 was done after 8 min centrifugation at 1800 rpm.

### Cytokine-induced killer cell generation

The blood (donations from UKB) was mixed with PBS (1x) containing 1% bovine serum albumin (BSA) (Life Technologies; PAA, Cölbe, Germany) and applied onto a Lymphoprep density gradient medium (Pancoll) (PAN BIOTECH, Aidenbach, Germany) in order to perform a density gradient centrifugation at 1,000 rpm for 30 min without breaks on. Afterwards the interphase containing the lymphocytes was removed and washed several times with PBS/1% BSA followed by treatment with an Ery-Lysis-Buffer. The extracted lymphocytes were then placed into a culture flask containing 40 ml of the modified RPMI medium and treated with 1,000 U ml^–1^ IFN-γ. One day after the extraction 300 U ml^–1^ IL-2, 50 ng ml^–1^ α-CD3 and 100 U ml^–1^ IL-1β were added to the cells. Every third day half of the medium was exchanged and 300 U ml^–1^ IL-2 were added. The CIK cell generation is complete after two weeks of maturation.

The generated Cytokine-induced killer cells were cultured in RPMI 1640 medium with L-glutamine and 2.0 g l^–1^ Sodium bicarbonate containing 10% FBS, 100 U ml^–1^ P/S and 2.5% hepes. The CIK cells were stored under the same conditions as ccRCC cells but were passaged every three days followed by the addition of 300 U ml^–1^ human interleukin 2 (IL-2).

### Chaetocin preparation

Chaetocin (Santa Cruz Biotechnologie, Heidelberg, Germany) was dissolved in high concentrations with DMSO. The stock solution was kept at –20°C for a maximum of one month. Prior to the experiments the stock solution was diluted with the corresponding medium.

### MTT assay

The cell lines were applied onto a 96-well plate with a concentration of 1 * 10^5^ cells ml^–1^ in the presence of different chaetocin concentration in the range of 1 nm to 400 nm. 

The chaetocin was first solved in DMSO and afterwards diluted within the corresponding RPMI medium. The cells were then incubated for 1 to 4 days without further addition of chaetocin during the incubation.

After the incubation 3-(4,5-dimethylthiazol-2-yl)-2,5-diphenyltetrazolium bromide solved in PBS (MTT reagent) was added to the cells followed by another hour of incubation. 

The MTT reagent was removed and the cells were treated with DMSO to release the MTT reagent stored in the still living cells. The absorption of the released MTT was then measured with an ELISA-READER at 560 nM [9], [10].

### FACS analysis

The cancerous and non-cancerous cell lines were incubated along with CIK cells in a ratio of 1:10 in presence and absence of 200 nM chaetocin for 24 h. The chaetocin was first solved in DMSO and afterwards diluted within the corresponding RPMI medium. 

The cell suspensions were then treated with Hoechst 33258 staining solution (Thermo Fisher Scientific, Waltham, USA) for 10 min. 50,000 beads of Flow Cytometry Absolute Count Standard; Full Spectrum; 999,000 particles/ml (Bangs Laboratories Inc., USA) were added after additional washing. The stained cell solutions were then applied to the FACS (BD FACS CANTO II, BD Biosciences, Heidelberg, Germany). The program FLOWJO v10 OSX was used on an Apple Mac for data processing.

### Statistical analysis

The statistical analysis was performed using Microsoft Office Excel 2010. Triplicates were used for every assay to determine the mean and ±SD. The significance was determined by the use of an unpaired two-sample location t-test (*=0.05–0.04, **=0.02–0.03, ***=<0.002).

## Results

### Chaetocin titering

**Figure 1:**
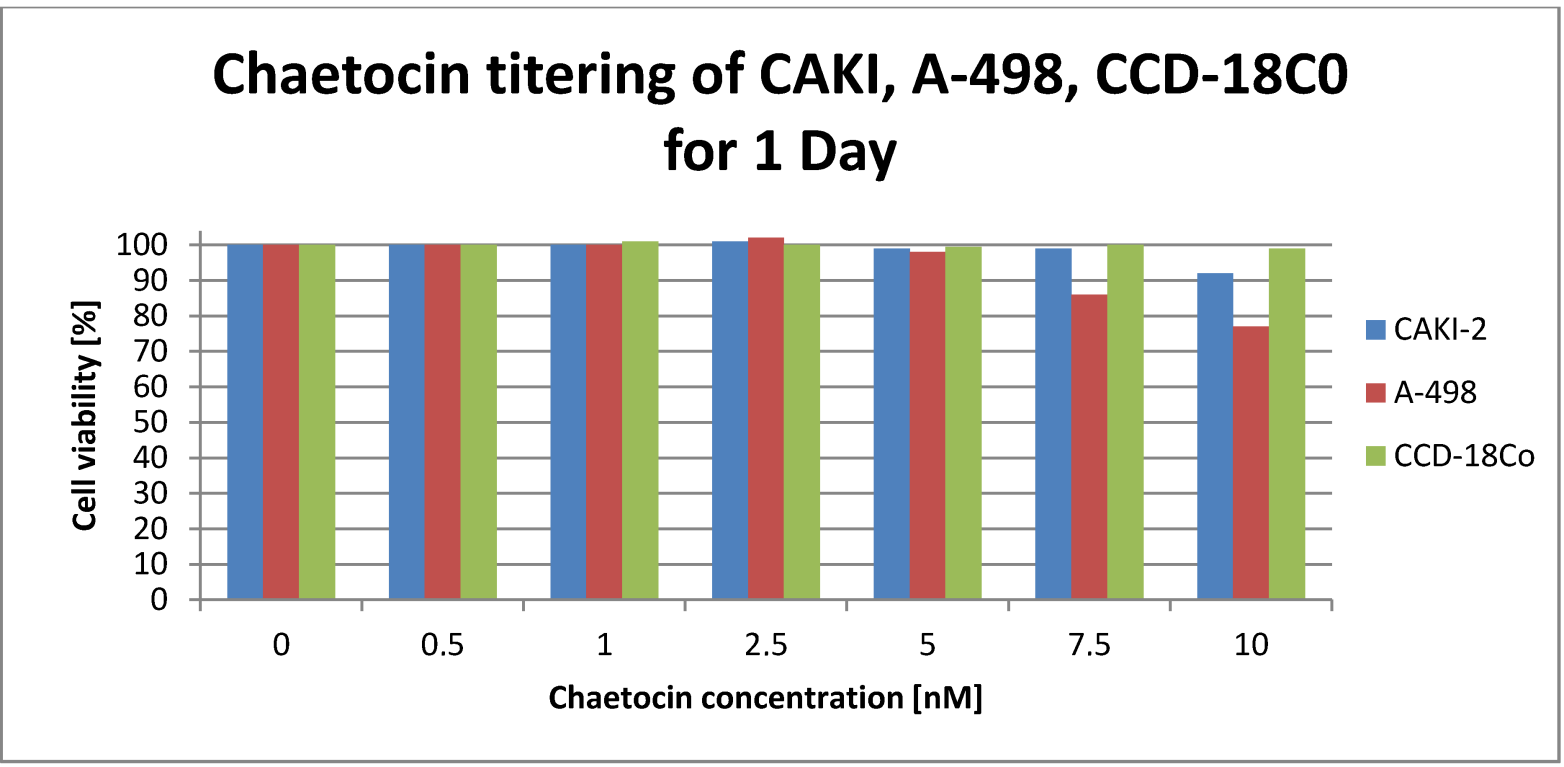
Viability of tumor cell lines CAKI-2 (blue, left), A-498 (red, middle) and non-cancerous cell line CCD-18Co (green, right) after 24 h of exposure to chaetocin concentration in the range of 0–10 nM determined with a micro plate reader after MTT assay. Positive control; cell lines killed with DMSO. Negative control; cells without any treatment.

Prior research stated that 24 h exposure of 2–10 nM chaetocin cause the death of 50% of all tested solid tumor cell lines [11]. So the first step was a chaetocin tittering in the range of 0–10 nM with two tumor (A-498, CAKI-2) and one non-cancerous control cell line (CCD-18co) (see Figure 1 (Fig. 1)). The results of the MTT assay clearly show that the cell viability of CCD-18Co non-cancerous cell line is not affected by the chosen chaetocin concentration. The ccRCC cell line CAKI-2 shows a small drop in cell viability to 92% at a concentration of 10 nM chaetocin while the A-498 tumor cell lines viability drops to 86% at a chaetocin concentration of 5 nM and even to 77% at a concentration of 10 nM chaetocin.

### Chaetocin titering of ccRCC cell lines

**Figure 2:**
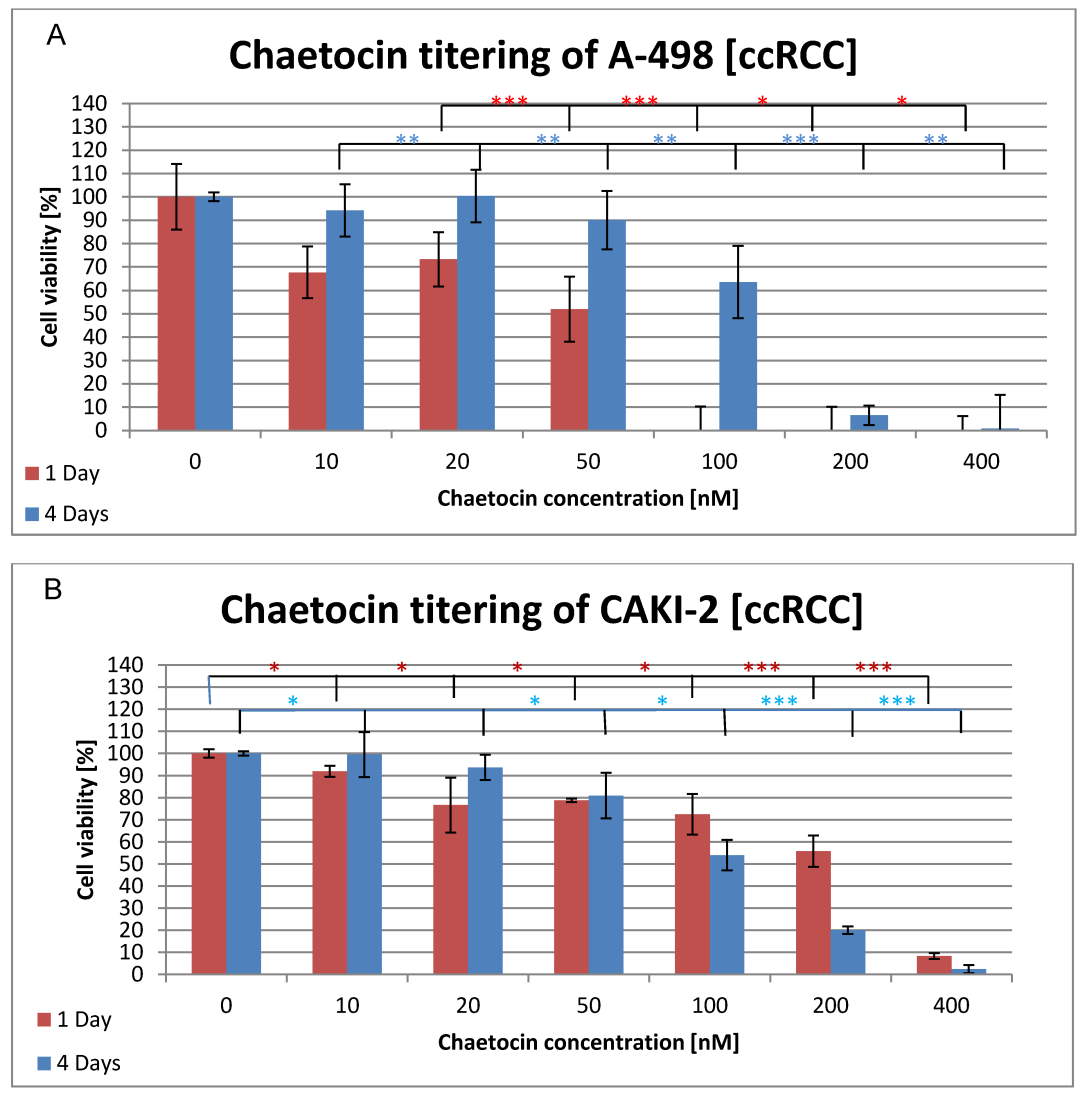
Cytotoxic effects of chaetocin on cell viability of ccRCC cell lines A-498 (A) and CAKI-2 (B). Both cell lines were incubated in medium containing 0–400 nM chaetocin and the cell viability was determined after 1 day (red) and after 4 days (blue) by the use of the MTT assay. Triplicates were used for each sample to determine the mean ±SD. The significance was determined by the use of t-test (*=0.05–0.04, **=0.02–0.03, ***=<0.002). Cells without chaetocin treatment were used as negative control while cells treated with a deadly dose of DMSO were used as positive control.

Since the chosen chaetocin concentrations showed a weaker effect upon the viability of the ccRCC cell lines than expected, higher concentration in the range from 0–400 nM were chosen for the next chaetocin tittering. The cell viability was determined after 1 day and 4 days of incubation, without additional administration of chaetocin, in order to see if the cytotoxic effect of the medical agent increases or if the cells are able to regenerate (see Figure 2 (Fig. 2)). Figure 2A (Fig. 2) shows the results of the chaetocin titering with A-498 cells. The cell viability of the A-498 cell line already dropped to 67% after one day of incubation with 10 nM chaetocin compared to A-498 cells grown in the absence of chaetocin. A further decrease of the cell viability is observed at a concentration of 50 nM where only 51% of the cells remain alive. No further signal of living cells was detected at concentration of 100 nM and above. The four day incubation of the cell line along with chaetocin showed quite different results. The cell viability stays above 90% until a concentration of 100 nm is reached which still resulted in a cell viability of 63% and 6% of the cells stayed alive even at a concentration of 200 nM chaetocin. Figure 2B (Fig. 2) displays the cell viability of the CAKI-2 tumor cell line during the chaetocin titering. The viability stays above 80% until a chaetocin concentration of 20 nM is reached resulting in a drop to 76% viability. Even at higher concentrations like 200 nM 55% of the CAKI cell line were still alive and 8% at 400 nM. After four days of incubation, the cell viability of the CAKI-2 cell line is above 90% until it drops to 81% at a concentration of 50 nM chaetocin. The results for the one day and four day incubation are quite similar for the concentration from 10 nM–50 nM but at 100 nM the viability of the four day incubated cells dropped to 55% and down to 20% at 200 nM.

### Chaetocin titering of a non-cancerous cell line

**Figure 3:**
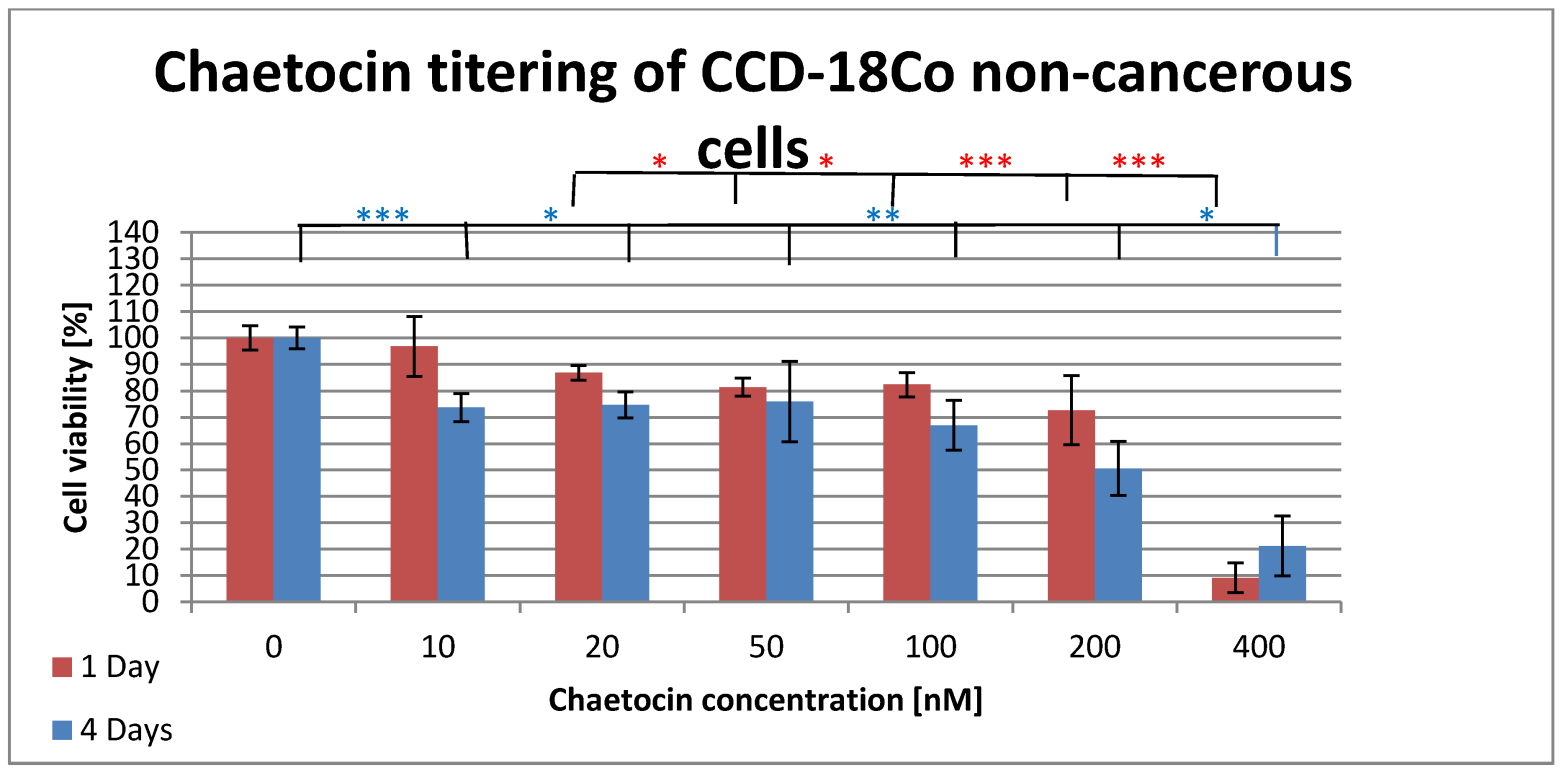
Cytotoxic effects of chaetocin on cell viability of non-cancerous cell line CCD-18Co. The cells were incubated in medium containing 0–400 nM chaetocin and the cell viability was determined after 1 day (red) and after 4 days (blue) by the use of the MTT assay. Triplicates were used for each sample to determine the mean ±SD. The significance was determined by the use of t-test (*=0.05–0.04, **=0.02–0.03, ***=<0.002). Cells without chaetocin treatment were used as negative control while cells treated with a deadly dose of DMSO were used as positive control.

CCD-18Co is a non-cancerous cell line originated from the human colon. This cell line is used to test the cytotoxic effects of chaetocin on non-cancerous cells in order to compare it with the effects on ccRCC cell lines. Figure 3 (Fig. 3) shows the results of the MTT test for non-cancerous colon cells that were incubated with different concentrations of chaetocin. The cell viability stays above 80% until a concentration of 200 nM is reached. Incubation with 200 nM chaetocin results in the death of 28% while a concentration of 400 nM kills 91% of the cells when incubated for 24h. Four days of incubation resulted in a drop of cell viability to 73% after administration of 10 nM chaetocin. The next drop in cell viability is observed at 100 nM where 66% of the cells were still alive while it was only 50% for 200 nM of chaetocin. The final concentration of 400 nM resulted in 21% cell viability which is more than twice the viability of the samples that were incubated for one day.

### Chaetocin titering of generated CIK cells

**Figure 4:**
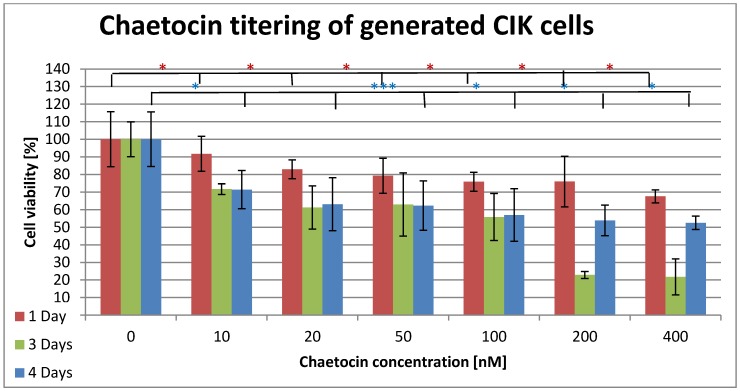
Cytotoxic effects of chaetocin on cell viability of generated CIK cells. The cells were incubated in medium containing 0–10 nM chaetocin and the cell viability was determined after 1 day (red), after 3 days (green) and after 4 days (blue) by the use of the MTT assay. Triplicates were used for each sample to determine the mean ±SD. The significance was determined by the use of t-test (*=0.05–0.04, **=0.02–0.03, ***=<0.002). Cells without chaetocin treatment were used as negative control while cells treated with a deadly dose of DMSO were used as positive control.

In order to use chaetocin in combination with CIK cells it must be ensured that the used chaetocin concentrations have minimal or no effects on the viability of the CIK cells. Figure 4 (Fig. 4) displays the effects of chaetocin on CIK cells when incubated up to four days with different concentrations. The cell viability decreased to 91% after one day incubation with 10 nM chaetocin. The decrease progresses slowly until a cell viability of 75% is reached at 100 nM and 200 nM. The final concentration of 400 nM still leaves 67% of the cells viable. After three and four days of incubation at a concentration of 10 nM chaetocin 71 % of the cells were still alive in both samples. The cell viability of both samples decreased slowly and steady to 56% at 100 nM. The incubation with 200 nM and 400 nM show the first difference between the three and four days of incubation because the cells that were incubated for three days only show a viability of 22% while the four days of incubation resulted in a viability of 53%. 

### Combination therapy 

**Figure 5:**
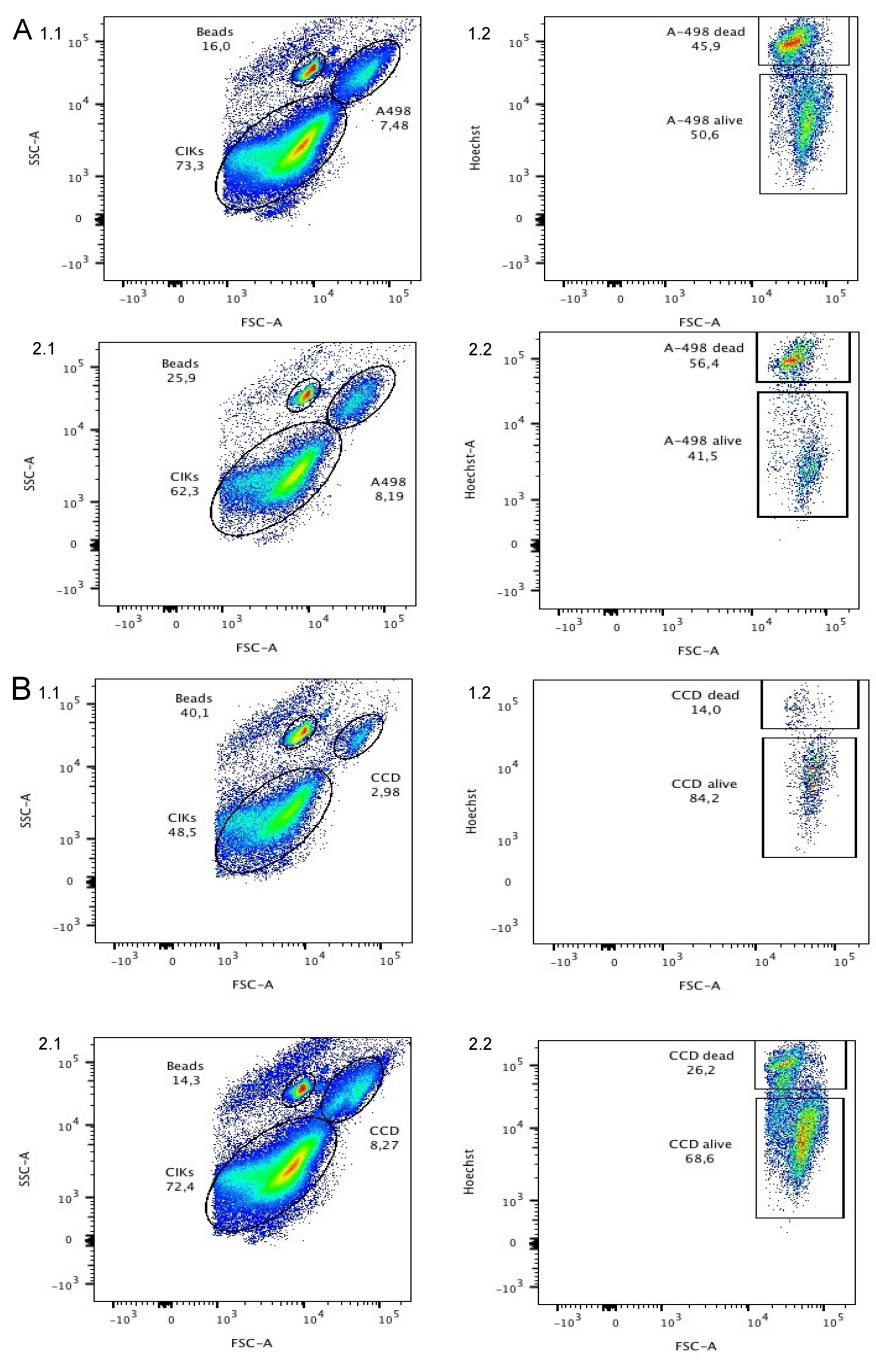
Results of the FACS analysis of ccRCC (A1, A2) and non-cancerous (B1, B2) cell line treated with CIK cells only (A1, B1) and CIK cells in combination with 200 nM chaetocin (A2, B2) for 24 h. Population of CIK cells and ccRCC cells were measured by FCS vs SSC. Hoechst 33258 stain and FSC was used to discriminate between living and dead ccRCC cells.

The ccRCC and CIK cells were combined in a ratio of 1:10 and incubated with and without chaetocin for 24 h. A concentration of 200 nM chaetocin was chosen since it killed 100% of the A-498 ccRCC cell line while only killing 28% of CCD-18Co and 25% of CIK. The MTT assay can be used to determine the cytotoxicity of a certain drug on a single cell line by simply stating how many cells survived the treatment. However, the MTT assay cannot be used to distinguish between two different cell lines when incubated together. In order to determine how many non-cancerous and ccRCC cells survived the incubation along with chaetocin and CIK cells a FACS was used. Forward scatter (FSC) and side scatter (SSC) were used to identify and discriminate CIK cells, non-cancerous and ccRCC cells. The Hoechst 33258 staining solution was used in order to stain dead cells independent of being cancerous or non-cancerous to see how many survive the treatment. It was only possible to test one non-cancerous and one ccRCC cell line due to a lack of resources. Figure 5A 1.1 (Fig. 5) shows the population of CIK cells and A-498 ccRCC cells after 24 h of incubation in the absence of chaetocin. Both populations could be discriminated by their SSC and FSC. Figure 5A 1.2 (Fig. 5) shows the ratio of dead and living A-498 cells discriminated by Hoechst vs FSC. After the treatment 50.6% of the ccRCC cells were still alive while 45.9 % died and 3.5% could not be matched to either group. Figure 5A 2.1 and A 2.2 (Fig. 5) show an identical experimental set up but this time the cells were incubated in the presence of 200 nM chaetocin. After 24 h of incubation along with chaetocin 56.4% of the ccRCC cells died while only 41.5% stayed alive and 2.1% could not be matched to either group. 

So the population of dead cells increased by 10.5% when chaetocin was added. Figure 5B 1.1 (Fig. 5) displays the population of CIK cells and CCD non-cancerous cells incubated together without chaetocin. Figure 5B 1.2 (Fig. 5) shows the ratio of dead and living CCD cells after incubation with CIK cells. After 24 h of incubation only 14% of the non-cancerous cells died while 84.2% stayed alive and 1.8% could not be matched to either group. The survival rate decreases by 12.2% to 68.6% as 200 nM chaetocin were added.

## Discussion

### Chaetocin titering of ccRCC, non-cancerous and CIK cell lines

Previous research stated that a concentration of 2–10 nm chaetocin caused the death of 50% of all tested solid tumor cell lines after 24 h of incubation while mostly sparing the non-cancerous cells [11]. So the first step was to recreate the conditions of these experiments to check if such a low concentration is already sufficient to kill 50% of the ccRCC cell lines. Therefore, two ccRCC and two non-cancerous cell lines were incubated with chaetocin concentration in a range of 0–10 nM (Figure 1 (Fig. 1)). After 24 h of incubation 99% of the CCD cells were still alive but also 92% of the CAKI 77% of the A-498 ccRCC cell line. This titering experiment was performed three times with two different chaetocin stock solutions in order to exclude the possibility that the used chaetocin is somehow inactive. Another titering experiment with higher chaetocin concentration had to be performed since a concentration of 10 nM was not enough to even kill 50% of the ccRCC cells. A range of 0–400 nM chaetocin was chosen in order to determine what concentration is suitable to kill at least 50% of the ccRCC cells while sparing non-cancerous cells. 

The ID_50_ of chaetocin for A-498 cells was reached at a concentration of 50 nM while a concentrating of 100 nM was high enough to kill 100% of all A-498 cells (Figure 2 (Fig. 2)). The CAKI cell line showed a quite different result since 200 nM chaetocin where necessary to kill 45% of the ccRCC cell line while even a concentration of 400 nM left 8.3% living cells (Figure 2 (Fig. 2)). Surprisingly the CAKI cells needed 4 times as much chaetocin as the A-498 cells to reach the ID_50_ and the A-498 cells still needed five times as much chaetocin as previously reported by other research groups. The non-cancerous CCD cells remained above 80% survival until a concentration of 200 nM chaetocin was reached, 400 nM still left 9.1% of the cells intact (Figure 3 (Fig. 3)). The second non-cancerous cell population was CIK cells that showed a survival of more than 75% at a concentration of 200 nM and 67% of the cells were even alive at 400 nM (Figure 4 (Fig. 4)). Even though the chaetocin concentrations needed to kill the ccRCC cells were much higher than expected, the effects were stronger on ccRCC than on non-cancerous cell lines. The reason why the CAKI cells survived much higher chaetocin concentrations than the A-498 cells is still unclear. The additional 4 days of incubation were performed in order to test if the cells are able to recover or if the cytotoxic effect increases along with higher incubation times. The A-498 cell lines showed recovery since 51% of the ccRCC cells died at a concentration of 100 nM after 24 h of incubation while 90% of the cells were alive after 4 days of incubation with the same amount of chaetocin. The CAKI and CCD cell lines showed different results since the cells incubated for 4 days showed a higher number of dead cells than ones incubated for 24 h. 75% of the CIK cells survived when incubated for 24 h while the survival dropped to 20% after 3 days but then rapidly increased up to 52% after 4 days of incubation. Due to a lack of resources only one ccRCC cell line could be subject to the combination therapy of CIK cells and chaetocin. Based on the MTT results, the A-498 cell line was chosen because of its higher susceptibility to chaetocin.

### Combination therapy of ccRCC and non-cancerous cell lines with CIK cells and chaetocin

The effects of CIK cells in combination with chaetocin on non-cancerous and ccRCC cell lines was tested although the cytotoxicity of chaetocin was not proven to be as strong and as selective as previously reported. Previous research had shown that a 1:10 ratio of CIK cells and ccRCC cells usually results in the death of 50% of the ccRCC cells after 24 h of incubation [8] and our work showed that 200 nm are sufficient enough to kill up to 100% of the A-498 ccRCC cell line. Based on this data ccRCC and non-cancerous cell lines were incubated in a 1:10 ratio along with CIK cells and 200 nM of chaetocin to test if the chaetocin increases the effect of ccRCC cell destruction along with CIK cells (Figure 5 (Fig. 5)). Again the cytotoxicity and selectivity of chaetocin were much lower than expected. The CIK cells alone were able to kill nearly 50% of the ccRCC cells within 24 h of incubation while only 14% of the non-cancerous cells died. A low number of non-cancerous cells are usually targeted and destroyed by CIK cells but the death of 14% of non-cancerous cell could also be due to the repeated washing and incubation steps during the cell preparation for the FACS analysis. Since the addition of 200 nM of chaetocin for 24 h resulted in the death of 100% of A-498 ccRCC cells according to the MTT results it is expected that all ccRCC cell should die during the incubation with CIK cells and chaetocin. The results of the FACS analysis state otherwise, the addition of chaetocin increased the number of dead ccRCC cells by only 10.5% but also increased the number of non-cancerous dead cells by 12.2%.

## Conclusions

Chaetocin acts by histone lysine methylase inhibition [12], [13]. It has been shown to be effective in human non-small cell lung cancer cells and in acute myelogenous cells [14], [15]. However, the cytotoxicity of chaetocin at low concentrations was in our experiments found to be much weaker than expected. Although identical culture and incubation methods and concentrations were used, the determined ID_50_ was more than 20 times higher than previously reported [11]. And even though non-cancerous cells were less affected by chaetocin than the ccRCC cells during the MTT assay, this could not be achieved in combination with CIK cells. During the combination therapy the number of dead non-cancerous cells increased stronger than the number of ccRCC cells while incubated with chaetocin and CIK cells in comparison to CIK cells alone. Based on these results chaetocin is not a suitable agent for treating ccRCC cells solely because the concentration needed to kill a sufficient number of ccRCC cells also caused the death of at least one fourth of the non-cancerous cells. As previously mentioned chaetocin accumulates in non-cancerous as well as in ccRCC cells and creates ROS but only causes the death of ccRCC cells because they cannot compensate the upcoming ROS concentrations [4]. It is reasonable that the high concentration of 100–400 nM caused such high ROS concentrations that also a part of the non-cancerous cells could not compensate by the use of the enzymatic systems. In addition the accumulation of such high chaetocin amounts alone might have killed the non-cancerous and cancerous cells to the same extent. The combination therapy showed that the addition of chaetocin increased the death of non-cancerous cells even more than the death of ccRCC cells and thus chaetocin is also not suitable for the combination therapy along with CIK cells. Nevertheless CIK cells worked exactly like expected. After 24 h of incubation nearly 50% of ccRCC cells were killed while only 14% of non-cancerous cells died. The anti-cancer activity along with the high *ex vivo* replication and easy generation allows the specific targeting of ccRCC cells while causing only few deadly side effects on the non-cancerous cells. 

The effects of chaetocin on other cancerous cell lines could be a subject of future experiments since it would be interesting to determine if the effects differ depending on the type of cancer cell. Also future studies should focus on understanding the exact mechanism of chaetocin’s assumed anti-cancerous activity.

## Notes

### Competing interests

The authors declare that they have no competing interests.
